# Editorial: Implication of oxidative, inflammatory, apoptotic and autophagy pathways in colitis

**DOI:** 10.3389/fphar.2023.1326176

**Published:** 2023-12-14

**Authors:** Hanan M. Hassan, Mohamed M. Salama

**Affiliations:** Department of Pharmacology and Biochemistry, Faculty of Pharmacy, Delta University for Science and Technology, Gamasa, Egypt

**Keywords:** inflammation, oxidative stress, autophagy, apoptosis, STAT3, ulcerative colitis

Ulcerative colitis (UC), is a chronic inflammatory disorder of the colonic mucosa and is characterized by repeated cycles of relapse and remission (Ungaro et al., 2017). It specifically affects the colon and rectum through multifactorial mechanisms associated with genetic alterations, environmental factors, microbiota, and mucosal immune dysregulation (Nakase et al., 2022). The classic presentation of UC include bloody diarrhea with or without mucus, rectal urgency, tenesmus, and variable degrees of pain that is often relieved by defecation. Histopathology is the definitive tool in diagnosing UC, assessing the disease severity, and identifying intraepithelial neoplasia (dysplasia) or cancer (Gajendran et al., 2019). UC is treated using 5-aminosalicylic acid, corticosteroids, thiopurines, and molecular targeted agents, depending on the extent and severity of disease (Kobayashi et al., 2020). Despite these revolutionary molecular targeting therapies introduced during the last two decades, 30%–55% of patients fail to respond to such molecular targeting agents in the induction phase, requiring changes in treatment (Nakase et al., 2022).

Efforts to understand the large proportion of primary non-responders to each molecular targeted agent require in-depth knowledge of the molecular mechanisms of the pathology of UC. An impressive body of experimental and clinical evidence shows that the development and progression of UC involves multifactorial processes, mainly characterized by dysregulated immune responses and epithelial barrier defects (Ungaro et al., 2017), which may culminate in progressive damage and insufficient repair of the gastrointestinal tract. Thus, intensive investigations have focused on immune cells and soluble ligands, including cytokines, as therapeutic targets (Tong et al., 2021).

The complex pathology of UC may induce differences in responses to therapy. The findings of such studies strongly support the argument that future targeted therapies must focus on differences in cytokine levels associated with the stages of UC as well as on the distinct cytokine expression profiles of individual patients.

The Research Topic *Implication of oxidative, inflammatory, apoptotic and autophagy pathways in colitis* aims to provide a concise overview of the important molecular mechanisms and their interactions in the development and treatment of colitis.

The article by Zhang et al., titled *Bacteroides fragilis strain ZY-312 facilitates colonic mucosa regeneration in colitis via motivating STAT3 signaling pathway induced by IL-22 from ILC3 secretion* sheds light on that the intestinal epithelial barrier impairment contributes to the amplification of the inflammatory bowel disease (IBD)-associated immuno-inflammatory response and the imbalance of intestinal microbiota. Besides, it is currently evidenced that probiotics attract attention for its therapeutic effects in promoting intestinal epithelial barrier restoration, inhibiting immunity-mediated inflammation reaction, and modulating intestinal microbiota (Chopyk and Grakoui, 2020). Hence, the authors found that targeting epithelium barrier is considered a good candidate for IBD therapy.

**FIGURE 1:**
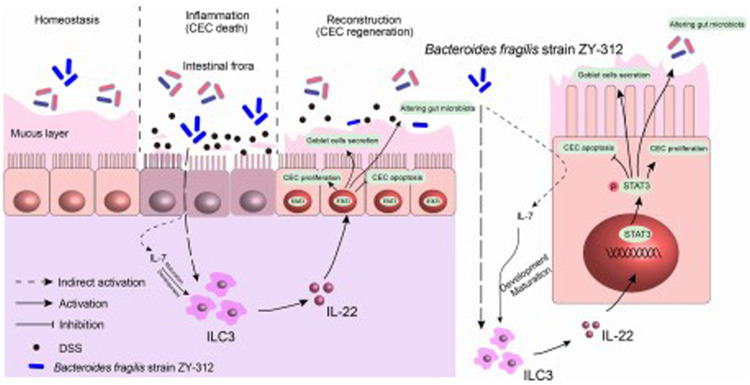
*B. fragilis* facilitates colonic mucosa regeneration in colitis via motivating the STAT3 pathway induced by IL-22 from ILC3 secretion. *B. fragilis* promoted CLP-derived ILC3 to secrete IL-22, then motivated the STAT3 signaling pathway, hence promoting colonic mucosa proliferation and mucus secretion, inhibiting apoptosis, and altering gut microbiota in DSS-induced colitis.

This study provides evidence that *B. fragilis* elicits positive effects on IBD by promoting the regeneration of the colonic mucosa through innate immune system mechanisms. Furthermore, IL-22 derived from ILC3 cells, has a crucial role in permitting *B. fragilis* to activate the STAT3 signaling pathway Figure 1. This activation facilitates the regeneration of the colonic mucosa by promoting cell proliferation, mucus secretion, and modulation of the intestinal microbiota leading to restoration of colonic mucosa. These findings open new possibilities for therapeutic interventions in colitis.

The article *Unveiling the therapeutic potential of exogenous β-hydroxybutyrate for chronic colitis in rats: novel insights on autophagy, apoptosis, and pyroptosis* by Abdelhady et al. demonstrated the beneficial role of β-hydroxybutyrate (BHB) administration and a ketogenic diet (KD) in modulating chronic colitis induced by dextran sodium sulfate (DSS). The authors found that BHB administration and consuming a KD effectively suppressed the NLRP3 inflammasome signaling, including the priming signal mediated by NFκB. In addition, they confirmed the restoration of redox homeostasis in the colon, evidenced by the decrease in ROS and MDA levels, along with a significant increase in GSH and SOD levels. Moreover, the administration of BHB or a KD resulted in a significant decrease in the elevated levels of inflammatory cytokines, such as TNF-α and IL-6, likely due to the inactivation of NFκB. As well, the inhibition of NLRP3 led to the downregulation of the active forms of IL-1β and IL-18, due to the repression of caspase-1 activity in conjunction with NFκB inhibition. Besides, both BHB and KD regimens exhibited the ability to attenuate caspase-3 activation, indicating their antiapoptotic potential. The current work verified that both BHB and KD have the potential to modulate UC by induction of three tight junction proteins: ZO-1, OCLN, and CLDN5 (Jiang and Wang, 2013), that play crucial roles in maintaining the integrity of the intestinal barrier that exert a protective effect on intestinal function. Indeed, this study provides compelling evidence that exogenous BHB administration in chronic colitis leads to the restoration of colonic tissue integrity and amelioration of inflammation. In time, the translation of these findings into clinical practice holds promise for improving the treatment outcomes of patients suffering from chronic colitis.

With a specific focus on the case report *Exploring Teduglutide as a Therapeutic Option for Refractory Microscopic Colitis: Insights and Implications*, the author Rim et al., present a patient who was diagnosed with lymphocytic colitis, a subtype of microscopic colitis characterized by ≥ 20 intraepithelial lymphocytes per 100 surface epithelial cells. His small intestine was affected, with blunted villi and lymphocytic infiltration which could be additionally contributing factors to his diarrhea. The patient was previously treated with multiple regimens, including budesonide, 6mercaptopurine, and infliximab. However, after the treatment with teduglutide, a glucagon-like peptide-2 (GLP-2) analog, the patient experienced not only nutritional conditions improvement but also showed enhancement in the microscopic colitis itself. The authors hypothesized that the alleviation of symptoms is due to the intestinotrophic effects of GLP-2 as GLP-2 is known to exert potent anti-inflammatory and anti-apoptotic effects in the gastrointestinal tract (Sigalet et al., 2007). GLP-2 may cause substantial drop in gene expression of tumor necrosis factor-alpha and interferon-gamma (Alavi et al., 2000) or might ameliorate myeloperoxidase activity, cytokine induction, and apoptosis resulting in protecting the integrity of the mucosal epithelium (Boushey et al., 1999) hence reversing the flattening and degeneration of epithelial cells in microscopic colitis. The study provides a valuable understanding of the complex molecular pathways involved in disease progression, nonetheless, continued investigations into the mechanisms of action and clinical trials evaluating the efficacy of teduglutide are warranted.

The article *Protein tyrosine phosphatase non-receptor type 2 as the therapeutic target of atherosclerotic diseases: past, present, and future* by Tang et al., discussed tyrosine-protein phosphatase non-receptor type 2 (PTPN2), as an important member of the protein tyrosine phosphatase (PTPs) family. PTPN2 is an intracellular PTP that consists of a PTP domain and a C-terminus domain that attracted more attention in recent years (Hongdusit and Fox, 2021). As a dephosphorylation enzyme, PTPN2 can negatively regulate many signaling pathways through dephosphorylation. The biggest manifestation is that PTPN2 can inhibit multiple inflammatory signaling pathways (Meng et al., 2019). PTPN2 mainly inhibits the occurrence and development of diseases by negatively regulating the expression of downstream target genes and their signaling pathways. These target genes are involved in a series of inflammatory responses, which in turn affect the function of vascular endothelial cells (VECs), monocyte proliferation and migration, macrophage polarization, T cell polarization, autophagy, pyroptosis, and insulin resistance, and may play an important role in the disease progression of atherosclerosis. The authors reported that ABCA1 is a key protein in the reverse cholesterol transport process, which can promote macrophages’ excretion of lipids, thereby inhibiting the development of atherosclerosis. This could be explained according to (Hao et al., 2009) who reported that in macrophages, IFN-γ downregulates the expression of ABCA1 by activating the JAK/STAT1 signaling pathway, thereby promoting the development of atherosclerosis. On the other hand, PTPN2 in macrophages regulates IFN-γ, JAK/STAT1, IL-4/6, and NF-κB-induced inflammation. More work is needed to elucidate how PTPN2 can be most efficiently targeted through transcriptional/post-transcriptional regulation or post-translational modification.
